# Editorial: RNA Modification in Human Cancers: Roles and Therapeutic Implications

**DOI:** 10.3389/fgene.2022.845744

**Published:** 2022-01-31

**Authors:** You Zhou, Tao Huang, Tianbao Li, Jing Sun

**Affiliations:** ^1^ First People’s Hospital of Changzhou, Changzhou, China; ^2^ Shanghai Institute of Nutrition and Health, Chinese Academy of Sciences, Shanghai, China; ^3^ Geneis (Beijing) Co. Ltd., Beijing, China; ^4^ Department of Pathology, George Washington University, Washington, DC, United States

**Keywords:** RNA modification, epigenetics, sequencing technologies, human cancers, gene regulation

Rapid advances in high-throughput sequencing technologies have revolutionized our understanding of the mammalian DNA and RNA. The recently identified RNA modification has emerged as a new layer of regulatory mechanism controlling gene expression in human cancers. Moreover, these epigenetic modifications are found not only in messenger RNA but also in non-coding RNA, adding additional complexity to the RNA world. Non-coding RNAs consist mainly of microRNA (miRNA), long non-coding RNA (lncRNA) and circular RNA (circRNA) (Quinn and Chang, 2016). As the most prevalent internal RNA modification in eukaryotes, N^6^-methyladenosine (m^6^A) has been reported to not only regulate stability and degradation of non-coding RNA itself, but also participate in the pathogenesis of cancers by regulating cell proliferation, metastasis and homeostasis (Liu et al., 2018; Ma et al., 2019; Zhang et al., 2020). Accumulating research has witnessed the high frequency of m^6^A modification in cancers which could be used to predict diagnosis and prognosis of cancer patients (Chen et al., 2018). However, the details of the mechanism and the heterogeneity of how RNA modification influences cancer development and prognosis are still unknown. And targeting dysregulated RNA modification regulators represents an attractive strategy for cancer therapy. This research topic aimed to explore the roles and therapeutic implications of RNA modification in human cancers.

Recently, miRNA and lncRNA have been emerging as key regulators of gene expression in carcinogenesis (Bhan et al., 2017; Saliminejad et al., 2019). Lu et al. uncovered that miR-27a-3p modulated ferroptosis via targeting SLC7A11 in non-small cell lung cancer cells, implying the importance of miR-27a-3p/SLC7A11 in ferroptosis. Zhang et al. constructed a nine-miRNA risk signals associated with esophageal cancer prognosis and further bioinformatics analysis revealed several key signaling which could guide decision-making in clinic. By comparing differentially expressed miRNAs in metastatic melanoma with primary melanoma, Gao et al. identified three miRNAs which participated in melanoma metastasis through regulating their target genes. Du et al. revealed that overexpression of miR-1-3p significantly inhibited colorectal cancer cell proliferation and metastasis by restraining YWHAZ-mediated epithelial-mesenchymal transition. Ku et al. automated the acoustic microfluidics-based extracellular vesicles enrichment technique for small RNA sequencing and identified several miRNAs with potential diagnostic value in prostate cancer. Han et al. reviewed the fundamental biology and characteristics of exosomal lncRNAs as well as their functions between cancer cells and non-cancer cells. Shao et al. showed that exosomes from adipose-derived mesenchymal stem cells mediated lncRNA-MIAT alleviation of endometrial fibrosis via regulating miR-150-5p. Wang et al. indicated the sponge function of lncRNA FIRRE in promoting gallbladder cancer progression by regulating miR-520a-3p/YOD1 axis. Liu et al. discovered a novel androgen-induced lncRNA FAM83H-AS1 which stimulated prostate cancer progression through miR-15a/CCNE2 axis. In ovarian cancer, lncRNA NEAT1 was recognized by Jia et al. as a poor prognosis risk and induced chemotherapy resistance via miR-491-5p/SOX3 signaling pathway. Li et al. uncovered pharmacological mechanisms of Ketamine in suppression of ovarian cancer cell growth by controlling lncRNA-PVT1/EZH2/p57 axis. Through integrating advanced machine learning methods including Monte-Carlo feature selection, Incremental Feature Selection (IFS) and Support Vector Machine (SVM), Xia et al. discovered blood lncRNA signature associated with hepatocellular carcinoma progression. Niu et al. established a risk score model based on gene signature of multiple survival-associated differentially expressed genes and identified a five-lncRNAs signature to predict herapeutic efficacy and prognosis in glioblastoma. Chen et al. uncovered anti-tumor effects of Ginsenoside Rh7 through targeting ILF3-AS1-mediated miR-212/SMAD1 axis in non-small cell lung cancer.

Organized as a covalently closed loop, circRNA can modulate gene expression in various stages of tumor development (Kristensen et al., 2019). One of most widely studied mechanisms refers to its sponge function (Panda, 2018). Several studies in our topic elucidated this effect in various cancers. Ghafouri-Fard et al. reviewed the oncogenic role of circPVT1 which acted as sponges for dozens of miRNAs including miR-125a, miR-125b and miR-124-3p, among different cancers based on experimental and clinical investigations. Yang et al. revealed that circHIPK3 facilitated gastric cancer progression through miR-637/AKT1 pathway. Through analyzing gene expression profile of atherosclerosis, Kang et al. identified the function of circHIPK3/miR-637/CDK6 axis in stimulating cell proliferation in human vascular smooth muscle cells. Zhang et al. demonstrated the role of circDENND2A in accelerating non-small cell lung cancer progression through miR-34a/CCNE1 signaling. Liu et al. emphasized the importance of circHIPK3/miR-124/CCND2 axis in gliomagenesis.

Increasing studies have investigated m^6^A modification on the biological functions involved in cancer progression and our topic also collected these findings (Liu et al., 2018; Zhang et al., 2020). Qu et al. summarized the mechanism of m^6^A-related regulators as well as their potential therapeutic value in hepatocellular carcinoma. Tan et al. systematically conducted data analysis and identified key m^6^A-related lncRNAs in colon adenocarcinoma. Guo et al. built a prognostic model of hepatocellular carcinoma in order to discover m^6^A-related lncRNAs engaged in immune infiltration. Chen et al. demonstrated that GLS2 was regulated by METTL3 and METTL3/GLS2 signaling accelerated esophageal squamous cell carcinoma metastasis. Wei et al. globally investigated the genetic landscape of the m^6^A regulators in ovarian cancer and suggested their associations with clinical prognosis. Wang et al. found that m^6^A reader YTHDF1 aggravated the progression of cervical cancer by regulating its target gene RANBP2. Cheng et al. showed elevated m^6^A level for RNA methylation as well as METTL3 expression level in diffuse large B-cell lymphoma. Further analysis suggested METTL3 promoted cancer progression by regulating m^6^A level of pigment epithelium-derived factor.

Several studies also identified novel tumor promoter and suppressors in a variety of cancers. Annexin family proteins were identified and analyzed by Wu et al. that had prognostic value in bladder cancer and could be used as biomarkers for subtype classification. Liu et al. revealed the new function of PDK4 in modulating glucose metabolism as well as promoting gastric cancer progression. Gu et al. investigated the prognostic value and immune infiltration patterns of hippo pathway core genes in lung squamous cell carcinoma, contributing to precisely selection of patients with immunotherapy benefits in clinic. Gao et al. identified FoxM1/AKR1C1 axis as novel target of avasimibe by forming a positive regulatory loop, providing an alternative therapeutic option for cholangiocarcinoma. Hao et al. comprehensively analyzed aerobic exercise-related genes and found that CDCA4 depletion largely hinder osteosarcoma cancer progression. Through analysis of macrophage-regulated genes, Qi et al. indicated the tumor-promoting effects of PSMA2 in colorectal cancer. Using three separate datasets, Zhu et al. demonstrated key exercise-induced genes including ITGB2, WDFY4 and CYBB as well as corresponding pathways, implying their potential prognostic value in malignant melanoma. Hua et al. identified nine RNA binding protein genes through bioinformatics analysis and constructed a prognostic model based on these genes, exhibiting good efficiency in predicting patient survival of prostate cancer. Similarly, a study in hepatocellular carcinoma conducted by Huang et al. identified an RNA binding protein-related six-gene prognostic signature.

Other cancer risk factors were also investigated in this topic. Chen et al. found smoking quitting should be one of therapeutic methods for smoking-related squamous cell carcinoma. They also provided a risk score model to predict prognosis and select beneficial patients. Yang et al. developed a deep convolutional neural network-based framework to evaluate efficacy of immunological therapy for lung cancer from histopathological images. Huang et al. also proposed deep learning techniques to identify mutant genes for target-drug therapy in clinical practice of lung cancer. Li et al. systematically depicted the landscape of *cis*-and *trans*-acting expression quantitative trait loci of lncRNAs in human cancers and assessed its impacts on cancer immunity and treatment. Through single-cell RNA-seq analysis, Zhang et al. emphasized the crucial roles of humoral immunity infiltration and hepatocytic prognostic markers in hepatocellular carcinoma with cirrhosis. Through unsupervised clustering of T cell infiltrating levels of hepatocellular carcinoma patients, Li et al. evaluated their clinical significance and provided potential immunotherapeutic targets. Sun et al. also estimated immune infiltration status within microenvironment and identified CXCR4 and GPR183 as core genes for prostate cancer prognosis. Wang et al. developed a quantitative model to predict human age by integrating gene expression profiles from multiple tissues. Yang et al. reviewed the significant roles of circulating tumor DNA testing in diagnosis, tumor mutation burden, therapy response and clinical outcome prediction of non-small cell lung cancer. Gao et al. also reviewed current advance of application of liquid biopsy in metastatic colorectal cancer, especially in discovering new biomarkers. He et al. summarized advantages and disadvantages of *in silico* methods for repositioning drugs and chemical compounds in order to guide precise tumor treatment.
